# *Escherichia coli* Cytoplasmic Expression of Disulfide-Bonded Proteins: Side-by-Side Comparison between Two Competing Strategies

**DOI:** 10.4014/jmb.2311.11025

**Published:** 2024-03-29

**Authors:** Angel Castillo-Corujo, Yuko Uchida, Mirva J. Saaranen, Lloyd W. Ruddock

**Affiliations:** Faculty of Biochemistry and Molecular Medicine, University of Oulu, Oulu FI-90014, Finland

**Keywords:** CyDisCo, disulfide bonds, *E. coli*, recombinant protein production, SHuffle

## Abstract

The production of disulfide bond-containing recombinant proteins in *Escherichia coli* has traditionally been done by either refolding from inclusion bodies or by targeting the protein to the periplasm. However, both approaches have limitations. Two broad strategies were developed to allow the production of proteins with disulfide bonds in the cytoplasm of *E. coli*: i) engineered strains with deletions in the disulfide reduction pathways, *e.g.* SHuffle, and ii) the co-expression of oxidative folding catalysts, *e.g.* CyDisCo. However, to our knowledge, the relative effectiveness of these strategies has not been properly evaluated. Here, we systematically compare the purified yields of 14 different proteins of interest (POI) that contain disulfide bonds in their native state when expressed in both systems. We also compared the effects of different background strains, commonly used promoters, and two media types: defined and rich autoinduction. In rich autoinduction media, POI which can be produced in a soluble (non-native) state without a system for disulfide bond formation were produced in higher purified yields from SHuffle, whereas all other proteins were produced in higher purified yields using CyDisCo. In chemically defined media, purified yields were at least 10x higher in all cases using CyDisCo. In addition, the quality of the three POI tested was superior when produced using CyDisCo.

## Introduction

*Escherichia coli* is one of the most widely used organisms for the production of recombinant proteins in both academia and industry. This is due to its characteristics as an expression host: short duplication time, low cost of culturing, and ease of manipulation and scale-up [1]. For these reasons, *E. coli* has been used to express thousands of proteins, including one third of approved therapeutic recombinant products [2]. However, *E. coli* is limited by its inability to make many eukaryotic post-translational modifications (PTMs). These modifications are often essential for the proper folding and functionality of eukaryotic proteins. The most critical PTM for many proteins are disulfide bonds, which are found in roughly one-third of eukaryotic proteins [3, 4]. Formation of structural disulfide bonds is impossible in the reducing environment of the cytoplasm of wild-type *E. coli*.

Traditionally, two strategies have been used to produce proteins with disulfide bonds in *E. coli*. Recombinant proteins containing disulfide bonds can be produced either by ex vivo refolding from inclusion bodies or by translocation of the proteins into the periplasm. While oxidative refolding can be used on an industrial scale, it is often inefficient, highly protein dependent and usually requires extensive optimization for each protein [5]. In contrast, in the periplasm, disulfide bond formation occurs spontaneously due to the presence of the Dsb system [6]. However, the use of the periplasmic space has three limitations. First, the secretion apparatus of *E. coli* can be easily overloaded [7]. Second, the periplasmic space is limited, occupying approximately 16% of the whole cell volume [8]. Third, proteins that naturally fold in the periplasm have small numbers of disulfides and simple disulfide patterns, and the Dsb system often has problems with proteins that contain large numbers of disulfides or complex patterns.

The cytoplasm is the largest cellular compartment in *E. coli* and protein production here does not require export, and hence cytoplasmic expression removes two limitations of periplasmic expression. As such, two strategies have been employed to facilitate disulfide bond formation in the cytoplasm of E.coli: i) the use of engineered strains that remove reducing pathways; ii) co-expression of proteins that increase oxidative protein folding in vivo.

Wild-type *E. coli* strains have two pathways for disulfide bond reduction in the cytoplasm [9]. The first is based on the actions of thioredoxins (*trxA* / *trxC*), which are kept in the reduced state by the action of thioredoxin reductase (*trxB*). The second is based on the action of reduced glutathione (GSH), which is kept in the reduced state by the action of glutathione reductase (*gor*). Strains that have been engineered to remove reducing pathways usually knock out the *trxB* and *gor* reductases. As a result of these modifications, there is an increase in the spontaneous formation of disulfide bonds [10, 11]. Some examples of such strains are Origami (Novagen) and SHuffle (NEB). The lethality associated with these mutations is suppressed by a mutation in *ahpC*, which restores cellular viability [12]. At its simplest, native disulfide bond formation requires the action of an oxidase/oxidant, to oxidize a dithiol to a disulfide, and the action of an isomerase to rearrange non-native disulfides. The SHuffle strain incorporates co-expression of a cytoplasmic version of the normally periplasmic enzyme DsbC that catalyzes the later step. This has permitted the expression of a variety of proteins that contain disulfide bonds in their native state [11, 13].

The other strategy for cytoplasmic disulfide bond formation is the expression of proteins that catalyze oxidative protein folding. CyDisCo (cytoplasmic disulfide bond formation in *E. coli*) was the first [14, 15] and most widely used version of this strategy. CyDisCo has many formats, but the more common of these co-expresses two enzymes alongside the protein of interest (POI) to catalyze the two steps of native disulfide formation. Yeast mitochondrial thiol oxidase (Erv1p) uses molecular oxygen to oxidize dithiols into disulfides and human protein disulfide isomerase (PDI) isomerizes disulfide-bonded misfolded intermediates to their native state. CyDisCo has been used to express various difficult-to-express proteins of pharmacological and industrial interest [16-18], as well as to produce disulfide-containing proteins for structural studies [19-22]. It has been reported to produce proteins with up to 44 disulfide bonds [23]. Other variants of this strategy include the use of Erv2p or QSOX as the oxidase [24], or the use of specialist PDIs as the isomerase [25-27].

Despite the two strategies co-existing for more than a decade, to our knowledge the effectiveness of the two approaches has not been systematically evaluated. In addition, we are not aware of any proteins having been independently reported and expressed using the two strategies under conditions that would allow a direct comparison of the quantity or quality of the protein. In other words, there is no direct evidence as to what the relative abilities of the two systems are. Therefore, in this work we compared the two strategies side-by-side to evaluate the production of disulfide bond-containing proteins in the cytoplasm. The effect of commonly used promoters (Ptac or T7), strains (B or K-12 strain derived), and media (rich and defined autoinduction media) for the two systems was examined. The results showed that, by comparison to the strategy of removing reducing pathways, the strategy of adding catalysts for disulfide formation resulted in higher purified yields of POI, which require disulfide bond formation in order to be solubly produced. This effect was especially strong when using chemically defined media. In addition, for the three POI examined, the quality of the protein produced was better using CyDisCo.

## Materials and Methods

### Vector Construction, Plasmids, and Strains

BL21(DE3) with the CyDisCo system and SHuffle T7 Express were used for all B-derived strain tests. SHuffle T7 and MG1655 with the CyDisCo system were used in all the K12- derived strain tests. All the strains used are described in the supporting material (Table S1), and all the plasmids used for the POI are referenced as well (Table S2). The expression vectors of this study were generated by standard molecular biology techniques. The genes for mature *E. coli* alkaline phosphatase (PhoA) were amplified by PCR using a colony of *E. coli* XL1-Blue as a template, with added NdeI/BamHI sites in 5’ and 3’ respectively, and cloned into a modified pET23 vector with T7 and/or with Ptac promoter. The genes for hGH, chicken avidin, Angiopoietin-2 fibrogen domain and *Gaussia princeps* luciferase (Gluc) were synthesized codon optimized (co) for *E. coli* expression (GenScript). They were cloned NdeI/BamHI into one or both of the above- mentioned modified pET23 vectors. All plasmids generated were sequenced to ensure there were no errors in the cloned genes.

### Protein Expression and Purification

Plasmids carrying the gene of interest together with the CyDisCo plasmid pMJS205 (for plasmids with Ptac promoter) or pMJS226 (for plasmids with T7 promoter) when needed were transformed into the aforementioned strains using a heat shock transformation protocol. Selected transformant colonies were grown at 30°C in 2 ml of LB media supplemented with 2g/l glucose and the corresponding antibiotics (100 μg/ml of ampicillin for POI, 35 μg/ml chloramphenicol for CyDisCo plasmid), 250 rpm (2.5 cm radius of gyration) in deep well plates (DWP) covered with an oxygen-permeable membrane for 6-8 h as starter cultures. The starter culture plate was then used to seed another 24 DWP with either rich autoinduction media (Formedium) or defined autoinduction media [28] with 1:100 of the starter culture. Cultures with defined and rich autoinduction media were grown for 40 h and 24 h, respectively, in the same conditions as the precultures. OD_600_ was measured from the cultures and the cells were harvested by centrifugation at 3,220 ×*g* for 20 min at 4°C. The pelleted cells were resuspended in lysis buffer (40 mM Na_2_HPO_4_, 10 mM NaH_2_PO_4_, pH 7.4, 0.1 mg/ml egg white lysozyme, 20 μg/ml DNase), incubated for 15 min, and then frozen at -20°C. The cells were lysed using a freeze-thaw cycle once. Thawed lysates were centrifuged at 3,220 ×*g* for 20 min at 4°C, while the supernatant was collected and used for protein purification with HisPur Cobalt Resin (ThermoFisher) with gravity flow protocol as described earlier [16]. The eluted proteins were treated with 25 mM N-Ethylmaleimide (NEM) and run on SDS-PAGE under nonreducing conditions, as were untreated samples under reducing conditions. Page Ruler Plus Prestained Protein Ladder, 10 to 250 KDa (ThermoFisher) was used as the molecular weight marker. A linear concentration range of lysozyme standard was included on each gel and used for quantification of resolved POI concentration. This method allows us to quantify the concentration of purified POI without possible over-quantification due to contaminants or co-eluted proteins. Any observed co-eluted contaminants were excised and identified by matrix-assisted laser desorption ionization-time of flight-mass spectroscopy (MALDI-TOF MS) as described earlier [29].

### malPEG5000K Assay

To investigate the extent of disulfide formation, eluted protein samples for scFv Herceptin and Maa48 Fab were mixed with 0.1 M Tris buffer, pH 8, 0.1% SDS and 0.1 mM maleimide PEG (malPEG 5000, FLUKA). The reaction was covered from light and incubated for 40 min at RT. Immediately following incubation, non-reducing SDS-PAGE buffer was added to each tube, samples were heated at 95°C for 5 min, and then analyzed with SDS-PAGE.

### Gaussia Luciferase Activity Assay

For the luciferase activity assay, 20 μl of eluted Gluc purified from B-derived strains (derived from *E. coli* strain B) grown in rich or defined autoinduction media were mixed with 50 μl of working solution from the Pierce Gaussia Luciferase Glow Assay Kit (Thermo Scientific) according to the manufacturer's specifications. The amount of luminescence was measured after 10 min from the addition of a working solution using the Infinite M1000 Tecan reader.

### Data Processing and Analysis

A minimum of three replicates were used in all experiments. ImageJ software (https://imagej.nih.gov/ij/) was used to process and analyze all images of the gels in order to quantify the intensity of both POI and lysozyme bands. Data analysis and calculation were performed using the OriginPro 2019 software package (Originlab Corporation, USA). The lysozyme standards were fitted to a linear regression model to calculate the POI concentrations. Two-sample *t*-test comparisons were done between POI expressed by either CyDisCo or SHuffle in all data sets. All bar graphs represent the mean + standard deviation (SD). Significance levels were categorized as follows: ns = not significant; *p ≤ 0.05; **p ≤ 0.01; ***p ≤ 0.001.

## Results

### Selection of POI

In order for native disulfides to be formed in the cytoplasm of *E. coli*, either reductive pathways must be removed and/or oxidative pathways added. There are multiple variants for both strategies. We chose to use the SHuffle strain [11, 13] as an example of an approach where reductive pathways are removed as it adds also a catalyst of isomerization, the second step of native disulfide formation. As an example of adding oxidative folding catalysts, we chose to use the CyDisCo system [15, 16] as it was the first developed and specifically the Erv1p/PDI variant since it is the most widely used to date.

The first step of comparing these two approaches was to select the POI to be studied. To ensure that we did not include proteins that were impossible to fold via either system, our initial criteria required using POI that contain disulfide bonds in their native state and that were previously reported as having been successfully made in a biologically active form in one or both of the systems studied. However, early in this study it became apparent that some POI that were successfully made in these systems can be produced in a soluble state even in the absence of disulfide bonds, *e.g.* PhoA ([14], Fig. S1) or scFv Herceptin ([16], Fig. S1) or human growth hormone ([30], Fig. S1). This is problematic if the purpose of the study is to screen the relative ability of the systems to make disulfide bonds in proteins. To avoid this, an additional selection factor was added for subsequent POIs; specifically, that the POI was not solubly expressed in the cytoplasm of wild-type *E. coli*, *i.e.*, that the POI required disulfide bond formation to fold to a soluble state. The fourteen selected POI contain between one and five disulfide bonds, include POI with intermolecular disulfides, and have a range of sizes and protein folds (Table 1).

### Strain-Dependent Expression of POI

**Fig. 1:**
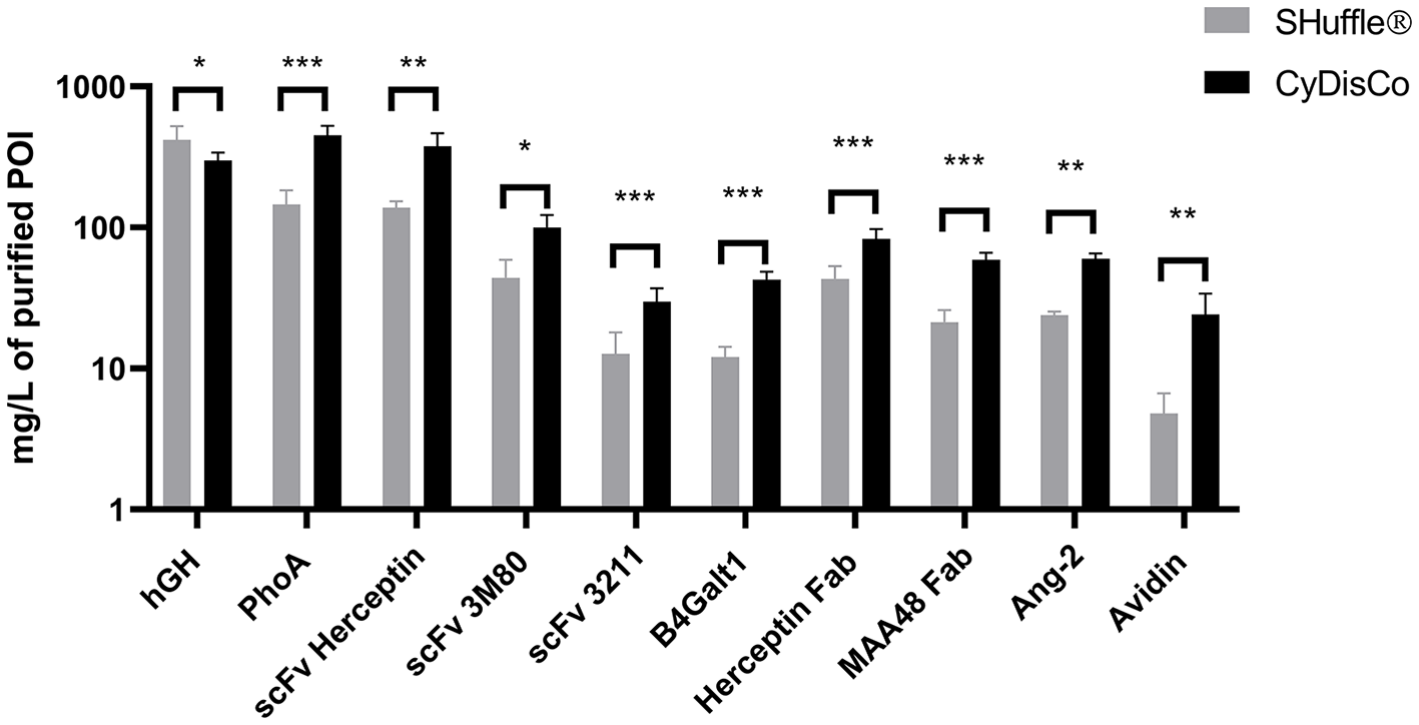
IMAC-purified yields of POI expressed in B strains using SHuffle and CyDisCo from a Ptac promoter in rich autoinduction media. The strains used were SHuffle T7 Express and BL21(DE3) +CyDisCo. Bars are mean + SD; *n* = 3-7 (see Table S3). *p* ≤ 0.05(*); *p* ≤ 0.01 (**); *p* ≤ 0.001 (***).

A selection of ten of these POI were then expressed under a Ptac promoter in *E. coli* B strains, either BL21 (DE3) with CyDisCo, or SHuffle T7 expressed in rich autoinduction media (see details of strains and plasmids used in Tables S1 and S2, respectively). Both systems were capable of producing all ten POI, with purified yields being POI and system dependent and ranging from 5-450 mg/l (Fig. 1). CyDisCo was able to produce nine of the ten POI in significantly higher purified yields than SHuffle, with hGH being the only POI produced in higher purified yields using SHuffle (Figs. 1, S2, and Table S3). This difference between the systems for disulfide formation was not due to higher cell densities as the mean OD_600_ at harvest was lower for 8 of the POI when using CyDisCo compared to SHuffle (Table S3).

**Fig. 2:**
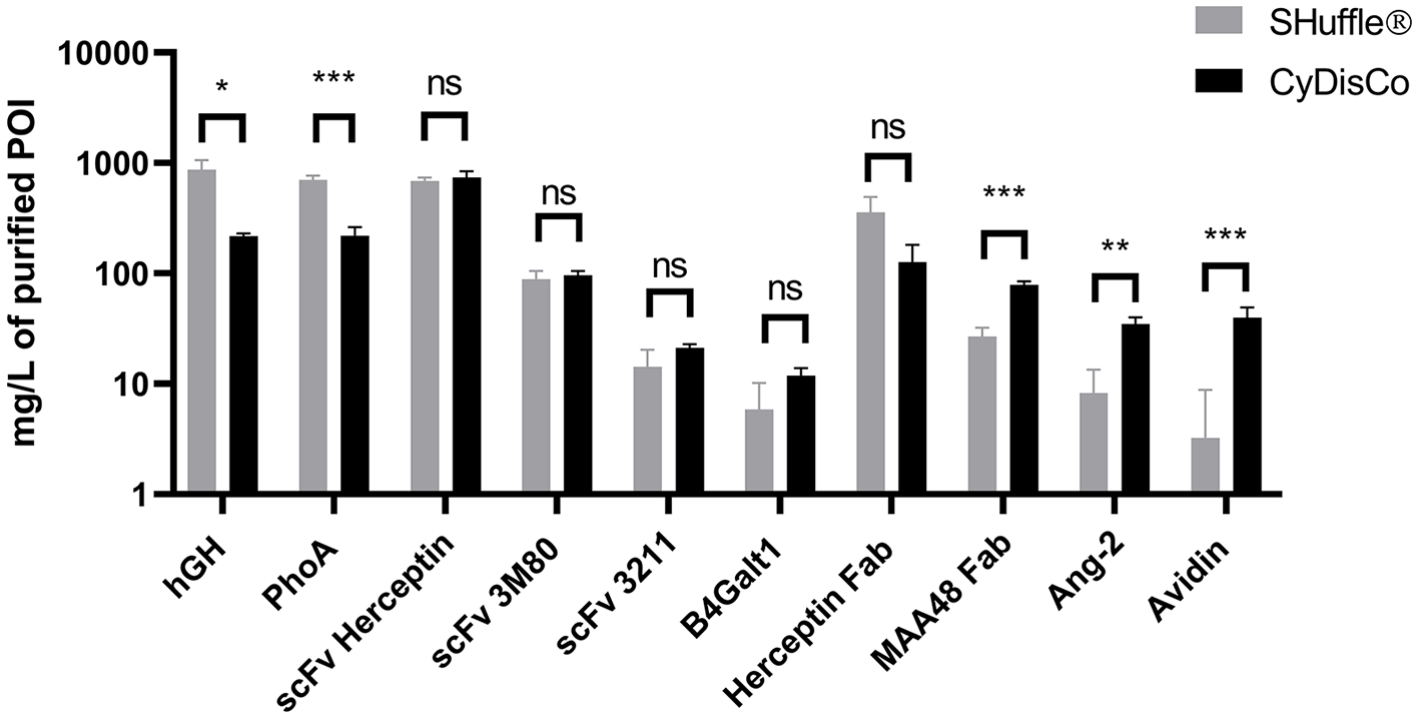
IMAC-purified yields of POI expressed in K strains using SHuffle and CyDisCo in rich autoinduction media. The strains used were SHuffle T7 and MG1655+CyDisCo. Bars are mean + SD; *n* = 3. Not significant (ns); *p* ≤ 0.05 (*); *p* ≤ 0.01 (**); *p* ≤ 0.001 (***).

*E. coli* K12-derived strains are widely used in industry. To examine potential differences in the effectiveness of the two strategies in K12 strains, expression in SHuffle T7 was then compared with POI expression in MG1655 with CyDisCo. Both systems could again produce all ten selected POI under a Ptac promoter in rich autoinduction media. In K12 strains, SHuffle produced two of the POIs in significantly higher yields while CyDisCo produced three of the ten POI in significantly higher yields (Figs. 2, S3, and Table S4).

Of the ten POI expressed in both K12 and B strain SHuffle, seven POI were purified in higher yields in the K12 strain (four significantly, Table S5) and three POI in lower purified yields (Tables S3 and S4). In contrast, purified yields using the CyDisCo system showed a reduction for six POI and an increase for four POI (one significantly, Table S5) upon expression in a K12 vs. B strain (Tables S3 and S4). It is difficult to dissect out where these POI-specific, strain-dependent effects arise from.

### Media Dependence

**Fig. 3:**
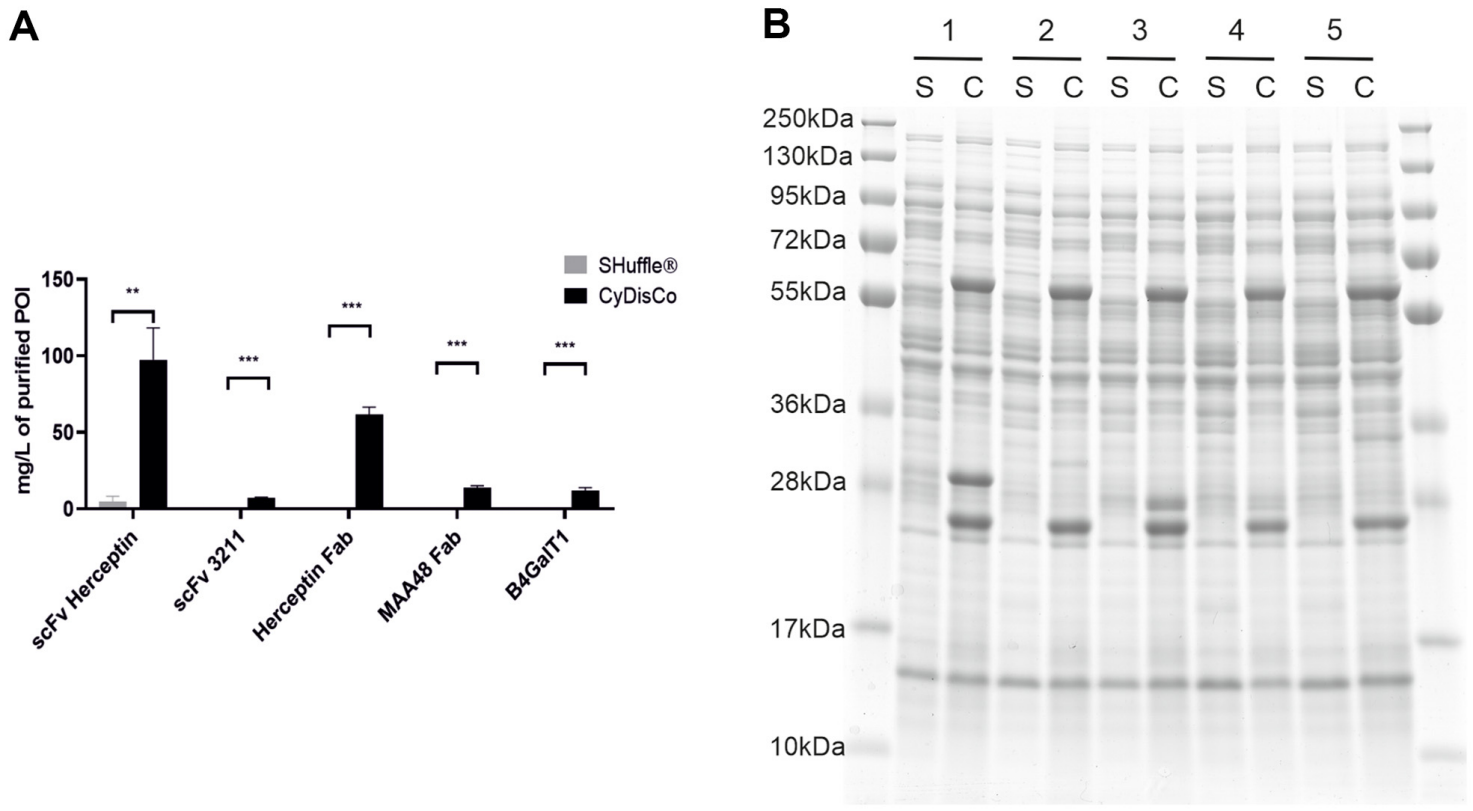
Protein production from B strains from a Ptac promoter in chemically defined media. (**A**) IMACpurified yields of POI. Bars are mean + SD (*n* = 3). *p* ≤ 0.01 (**); *p* ≤ 0.001 (***) (**B**) Reduced SDS-PAGE analysis of soluble cell lysates for POI produced using SHuffle (S) or CyDisCo (**C**). 1 ScFv Herceptin (27.2 kDa); 2) scFv 3211 (27.9 kDa); 3) Herceptin Fab (25.3 kDa, 23.6 kDa); 4) MAA48 Fab (24.7 kDa, 23.3 kDa); 5) B4GalT1 ((35.1 kDa). The strains used were SHuffle T7 Express and BL21(DE3)+CyDisCo.

Many types of media are used to produce recombinant proteins in *E. coli*. These can generally be grouped into two classes, rich media (which contain yeast extract or equivalent) and chemically defined media. To examine the media dependence of the two systems, five POI were expressed in B strains under a Ptac promoter in chemically defined media. All five POI could be purified in good yields using CyDisCo. In contrast, only one (scFv Herceptin) could be purified from SHuffle in detectable amounts and the yield for this POI was approximately 20x lower than with production using CyDisCo (Fig. 3A, Fig. S4, and Table S5). To ensure that this marked difference between the two systems was not solely due to differences in the IMAC purification efficiencies between the two strains in chemically defined media, cell lysates were examined by SDS-PAGE. All five POI produced using CyDisCo were visible in the SDS-PAGE, while those produced by SHuffle were not (Fig. 3B). Purified yields of all POI tested in both media were lower in the chemically defined autoinduction media than in rich autoinduction media; this in part comes from the lower harvest cell densities (Table S3 vs. Table S6).

### Promoter Dependence

**Fig. 4:**
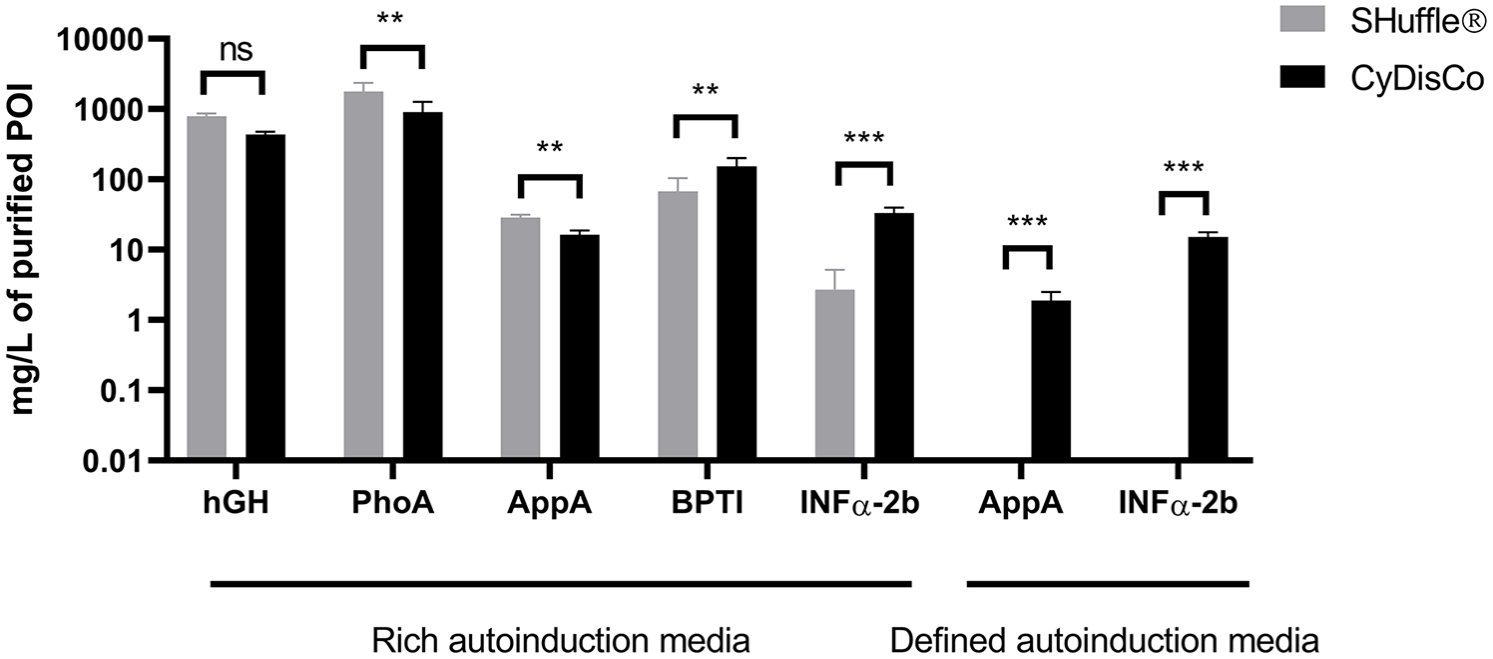
IMAC-purified yields of POI expressed in B strains using SHuffle or CyDisCo from a T7 promoter in either rich or chemically defined autoinduction media. The strains used were SHuffle T7 Express and BL21(DE3)+CyDisCo. Bars are mean + SD; *n* = 3-6 (see Table S7). Not significant (ns); *p* ≤ 0.01 (**); *p* ≤ 0.001 (***).

A wide range of inducible promoters are used for expressing recombinant proteins in *E. coli* [35] To determine whether the differences observed between the two systems for making disulfide bonds arose due to the promoter used, we tested the use of another promoter. Specifically, we selected the T7 promoter as: i) it is widely used in academia; ii) several SHuffle strains have been engineered to use it; iii) SHuffle is in part marketed in combination with T7 use. Five POIs were expressed in B strains in rich media using T7, and two of those were also expressed in defined media using the same promoter. Both SHuffle and CyDisCo systems were able to produce all five POI under T7 in rich autoinduction media, with the purified yields for three POI being higher using Shuffle, and the purified yields for two POI being higher using CyDisCo (Fig. 4, Fig. S5, and Table S7). In contrast, when chemically defined media was used, neither of the two POI tested could be purified from SHuffle in detectable amounts, while good yields of purified POI were obtained from CyDisCo expression (Fig. 4, Fig. S4, and Table S7).

### Quality of the Purified Products

The yield of protein produced is irrelevant if the POI is not correctly folded. To examine the presence of disulfide bonds, two POI which had been tested in all strains and gave good yields in rich autoinduction media for both systems were examined using reducing vs. non-reducing SDS-PAGE. Only material made in rich autoinduction media was examined since most POI expressed in chemically defined media gave no purified material when expressed in Shuffle, thereby preventing a comparison between the two systems.

**Fig. 5:**
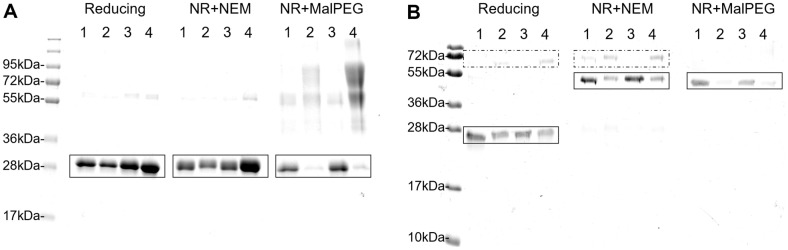
Quality of the purified scFv Herceptin and Maa48 Fab expressed under different conditions. SDS-PAGE of scFv Herceptin (**A**) and Maa48 Fab (**B**) expressed in either BL21(DE3)+CyDisCo (1), SHuffle T7 Express (2), MG1655+CyDisCo (3) or SHuffle T7 (4). Samples are analyzed as untreated under reducing conditions and as pretreated either with N-Ethylmaleimide or malPEG5000 under non-reducing conditions (NR+NEM or NR+malPEG, respectively). Box (solid line) surround the position of the POI under the different conditions, dotted box point a co-eluted contaminant. In this image, contrast and brightness have been modified to get better visualization of the bands. To see the original image, see Fig. S6.

ScFv Herceptin is a monomeric protein that contains two disulfide bonds in the native state. In the case of some proteins, the presence of disulfide bonds can be seen as a mobility shift of the protein band on reducing vs. non-reducing SDS-PAGE (with prior treatment by an alkylating agent such as N-ethylmaleimide (NEM) to ensure no thiol-disulfide rearrangement in the SDS) scFv Herceptin runs at the same position in both conditions (Fig. 5A). For such proteins, the addition of a high-molecular-weight alkylating agent, such as malPEG5000, allows examination of the disulfide state as its reaction with free cysteines in a POI results in an apparent shift in MW on SDS-PAGE. When treated with malPEG5000, the majority of the purified scFv Herceptin that had been expressed in SHuffle moved to higher molecular mobilities (Fig. 5A). This implies that the vast majority of the protein purified from SHuffle contains free cysteines, *i.e.*, it lacks disulfide bonds. This is consistent with the fact that this scFv can be made in soluble state in wild-type E.coli [16], *i.e.*, that its production is disulfide independent. In contrast, the majority of the scFv Herceptin expressed with CyDisCo showed no mobility shift upon addition of malPEG5000 (Fig. 5A), indicating that the purified protein had no free thiol groups and hence contained two disulfide bonds.

In contrast to scFv Herceptin, which is monomeric, Maa48 Fab is composed of heavy and light chains (app. 25 kDa each) linked by an inter-molecular disulfide bond. The presence of this intermolecular disulfide is easily visible by a mobility shift on reduced vs. non-reducing SDS-PAGE, from monomers to heterodimer, respectively. For both SHuffle- and CyDisCo- expressed Maa48 Fab, the majority of the purified protein ran at the dimer position on non-reducing SDS-PAGE, indicating that an intermolecular disulfide was present (Fig. 5B). However, the intensity of the SHuffle-produced protein in the non-reduced sample was lower than in the reduced, suggesting that other redox species were also present. Furthermore, the addition of malPEG5000 significantly reduces the intensity of the heterodimer band for the Maa48 Fab purified from SHuffle (Fig. 5B), implying the presence of free thiol groups, *i.e.*, incomplete disulfide formation. In contrast with CyDisCo-produced Maa48 Fab, the majority of the purified protein was unaffected by malPEG5000 treatment (Fig. 5B), indicating that it contained no free thiols, *i.e.*, that all cysteines were in disulfide bonds and that the heterodimer contained five disulfides.

In addition to observations from redox shifts, there are other indications relating to product quality for Maa48 Fab from the SDS-PAGE analysis. Specifically, the IMAC-purified Maa48 Fab, co-eluted with another protein, was clearly visible in both reducing and non-reducing conditions + NEM (Fig. 5B) with the amount relative to the POI being much greater for the Shuffle-produced Maa48 Fab than for the CyDisCo-produced protein. This protein was identified by MALDI-TOF MS as the molecular chaperone GroEL. Since GroEL is a chaperone which binds non-native proteins, this implies that a significant proportion of the Maa48 Fab produced in SHuffle is not native. In contrast, for CyDisCo-produced POI, the co-purifying GroEL band is either weak (B strain) or not visible (K12 strain) implying the Maa48 Fab produced was of higher quality. The GroEL band was also observed co-purifying with B4GalT1 (Fig. S7). With B4GalT1, protein purified from SHuffle strains has a higher intensity of GroEL bands than the POI, implying that most or all of the protein is non-native. In contrast, for the CyDisCo-produced POI, the intensity of the GroEL band was significantly lower than that of the B4GalT1, again implying the quality of the POI purified was higher using CyDisCo than that produced using SHuffle.

**Fig. 6:**
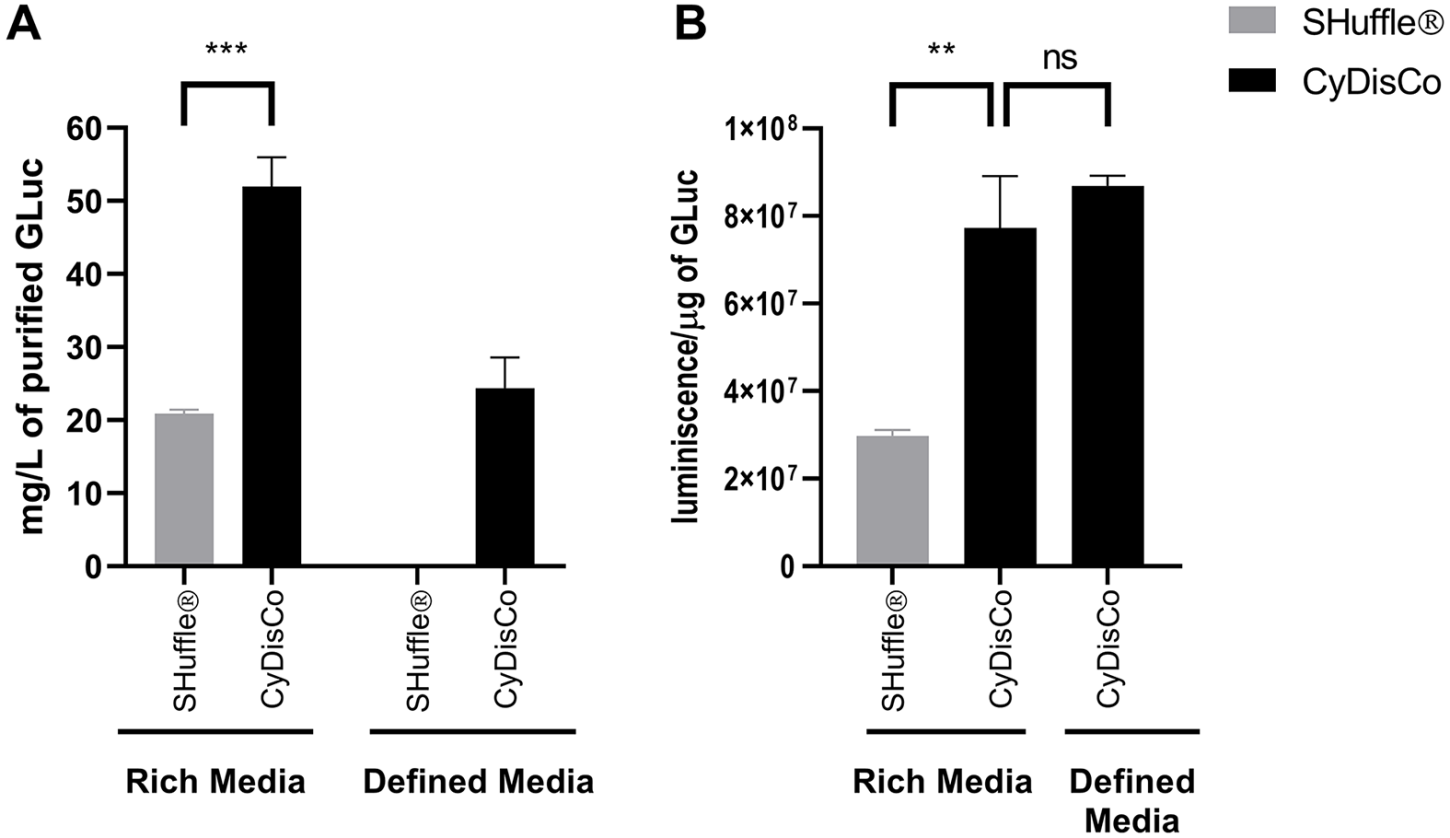
Purified yields and enzymatic activity of the GLuc expressed in B strains using SHuffle and CyDisCo from Ptac promoter in rich and chemically defined autoinduction media. (**A**) The calculated yields of Gluc expressed in SHuffle express and BL21(DE3) + CyDisCo. (**B**) Measured luminescence activity per microgram of purified Gluc protein. Note Gluc was not produced using SHuffle in defined media and therefore the activity could not be tested. Bars are mean + SD (*n* = 3). *p* ≤ 0.01 (**); *p* ≤ 0.001 (***).

Native disulfide bond formation is also linked to the activity of the protein, thus examining this is another way to assess the quality of the purified POI. One of the POI tested was an enzyme for which the activity can be easily measured, namely GLuc, which was expressed from Ptac in B strains in both rich and defined autoinduction media. In rich media, GLuc could be purified from both systems, with the yield of GLuc from the CyDisCo B strain being 3x higher than that from the corresponding SHuffle strain (Fig. 6A). The differences in purified yields between the media were in part dependent on the cell densities at harvest (Table S8). In contrast with chemically defined media, GLuc was purified only from the CyDisCo system, with no visible protein being purified from the SHuffle system (Fig. 6A, Fig. S8, and Table S8). This is consistent with other POI results in chemically defined media (Fig. 3, Table S6). By measuring luminescence-based luciferase activity, we found that purified GLuc expressed using CyDisCo had nearly 2.5x higher activity per microgram of purified protein than GLuc expressed using SHuffle (Fig. 6B). There was no significant difference in activity per microgram of purified protein between the GLuc expressed in CyDisCo in rich autoinduction media or chemically defined media, implying no media-dependent quality differences. These results demonstrate that not only do CyDisCo strains produce more GLuc, but they also produce POI of higher quality.

## Discussion

The production of recombinant proteins is a multi-billion-dollar market that involves different expression platforms, each with its own advantages and limitations. For many years, bacterial cell factories have been used to produce mainly proteins that either: i) do not require any posttranslational modifications; ii) require simple patterns of disulfide bond formation (via periplasmic expression); iii) can be recovered and refolded from inclusion bodies [36,37]. Two strategies have been developed to express disulfide bond-containing proteins in the cytoplasm of *E. coli* [38]. The first strategy, using engineered strains like SHuffle and Origami, is based on the disruption of reducing pathways normally present in the cytoplasm, while the second strategy involves co-expression of heterologous oxidative folding pathways. In this study, we undertook the first systematic comparison between examples of these two systems, in terms of purified yields of POI along with the quality and activity of some of the purified POI.

The use of purified yields as a comparison element between strains is a common approach in the biotechnology field to understand the overall relative productivity of different expression systems. Both systems tested here were able to produce multiple POI in high yields, suggesting both approaches are viable strategies. However, once the details are examined the results imply that CyDisCo may be the better system.

Some POI are produced in higher purified yields when using SHuffle than using CyDisCo or close-to-CyDisCo production levels in some strain/promoter combinations in rich media. However, all of these POI have been previously reported as being able to be produced solubly in the absence of a system for disulfide bond formation [6, 16, 39] (Fig. S1), despite the fact that all contain disulfide bonds in their native state. Hence, when no disulfide bond formation is required for soluble (non-native) protein production, yields in SHuffle using rich media can be higher than when using CyDisCo. This effect may arise since CyDisCo requires heterologous co-expression of oxidative pathway components; it has an increased metabolic burden (three proteins to be produced from plasmids, *e.g.* CyDisCo components + POI vs. just the POI). All other proteins were purified in higher yields from CyDisCo production and purified yields from chemically defined media of all POI tested were at least 20x higher from CyDisCo.

Proteins that can be made in a soluble state without native disulfides can also easily generate a mixed result in terms of the redox state of the POI when systems for disulfide formation are present. This is detrimental to the quality of the final product. For both POI examined, the purified protein produced using SHuffle had little protein that did not show a shift upon treatment with malPEG, implying that most of the protein did not have native disulfides. In contrast, most of the purified protein produced using CyDisCo showed no such mobility shift, implying it contained no free thiol groups. Redox heterogeneity is not the only indication that the POI expressed in SHuffle is of lower quality. Chaperone proteins such as GroEL [40-43] co-purifying with the POI (Maa48 and B4GalT1) indicates that at least a subset of the POI made in SHuffle are not correctly folded, while the lower enzymatic activity of GLuc produced via SHuffle also implies a higher level of correct folding with CyDisCo. Overall, CyDisCós engineered co-expression of oxidative folding pathways appear to make it superior to SHuffle in terms of both quantity and quality of purified protein.

A question then arises: why do systems that add catalysts of disulfide formation seem to be better than systems that have reducing pathways removed? There are two significant differences between the way SHuffle works compared to CyDisCo. The first is that SHuffle uses DsbC as the disulfide isomerase, while the CyDisCo variant we used (which is the most commonly used in the scientific literature) uses human PDI as the disulfide isomerase. For some POI this may be critical, but we do not believe this to be the primary factor for the difference in efficiency between the systems, especially as DsbC was used successfully as the isomerase in the first-reported CyDisCo system [14]. The second, and we believe the primary, difference between the two systems is how disulfide bonds are formed. In CyDisCo and equivalent systems, a sulfhydryl oxidase (Erv1p, Erv2p, QSOX etc) is used to oxidize dithiols in folding proteins to disulfides. These are active catalysts and form the primary route for disulfide bond formation in eukaryotes [44-46]. In contrast, in strains like SHuffle, the disruption of the thioredoxin- and glutathione-based reducing pathway functions lack an active dithiol-oxidizing mechanism. In these strains, thioredoxins are reported to work as catalysts of thiol-disulfide exchange [10]. However, they are not de novo disulfide bond formation catalysts. The way thioredoxins become reoxidized in these systems is probably by reducing other substrates, such as ribonucleotide reductase, and then transferring this disulfide to the folding POI. This makes disulfide bond formation in these strains a byproduct of other metabolic processes and possibly linked to the rate of DNA replication and/or to the amount of reactive oxygen species present. While these strains can produce more disulfide bond- containing proteins than wild-type *E. coli*, the purified yields and/or the quality of the products may be below optimal levels for use for many POI. In addition, their strong growth defect in some chemically defined media [47] and inability to make disulfide bond-containing proteins in chemically defined media decreases the possibility to use this engineered strain in many industrial processes. In contrast, the CyDisCo system can produce disulfide-bonded proteins in any context of media, promoter, and background strain. This, in our opinion, along with its catalyzed system for disulfide bond formation, makes it the more suitable option for the cytoplasmic expression of disulfide bonded proteins in *E. coli*.

## Conclusion

Both CyDisCo and SHuffle are capable of producing proteins that naturally have structural disulfide bonds in the cytoplasm of *E. coli*. Their distinct approaches, either the addition of a catalyst of disulfide bond formation or the removal of reducing pathways, is probably responsible for the difference in the results seen between them since both include a catalyst of disulfide bond isomerization. In addition to purified yields, the quality of the POI should also be examined to see if they are suited to the intended purpose, especially as some proteins are soluble with incomplete disulfide bond formation (such as scFv Herceptin or PhoA) in rich media. For proteins that require disulfide bonds to reach a soluble state, the quantity of POI and, for those proteins examined, the quality of the POI was better using a system that adds an active catalyst of disulfide formation. Hence, having active rather than passive systems for disulfide formation appears to be better.

## Supplemental Materials

Supplementary data for this paper are available on-line only at http://jmb.or.kr.



## Figures and Tables

**Table 1:** Proteins of interest (POI) used in this study with their respective sizes and number of disulfide bonds.

POI	Size (kDa)	Disulfide bonds	Ref.
Human growth hormone (hGH)	23.2	2	[17, 31]
*E. coli* alkaline phosphatase (PhoA)	47.5	2	[11, 14]
Humanized scFv Herceptin	27.2	2	[16, 32]
Human scFv 3M80	27.3	2	[16, 17]
Mouse scFv 3211	27.9	2	[16]
Humanized Herceptin Fab	48.8	5 (i)	[16, 18]
Human Maa48 Fab	47.9	5 (i)	[16, 18]
Human Beta-1,4-galactosyltransferase 1 (B4Galt1)	35.1	2	[33]
Human Angiopoietin-2 (Ang-2) fibrinogen domain	16.6	3	unpublished
Chicken Avidin	16.7	1	[17]
*Gaussia princeps* luciferase (GLuc)	19.2	5	[11, 34]
*E. coli* phytase (AppA)	42.4	4	[11, 14]
Bovine pancreatic trypsin inhibitor (BPTI)	7.60	3	[15]
Human interferon alpha 2b (IFNα-2b)	20.7	2	[15, 31]

Size marked includes an N-terminal hexa-histidine purification tag. (i) indicates that it includes an intramolecular disulfide bond. References refer to previously reported production, in the cytoplasm of *E. coli*.
