# Supplementary material for: Escherichia coli Cytoplasmic Expression of Disulfide-Bonded Proteins: Side-by-Side Comparison between Two Competing Strategies
Source: J Microbiol Biotechnol. 2024 Mar 29;34(5):1126–34. doi: 10.4014/jmb.2311.11025 (PMC11180911; doi:10.4014/jmb.2311.11025)
Supplement: Supplementary file 1 [file jmb-34-5-1126-supple.pdf]

Supplementary figure S1. Coomassie stained SDS-PAGE of POI expressed in both MG1655and BL21(DE3) using rich media. Non-reducing gel. Expressed using rich autoinduction media and Ptac promoter. Proteins expressed were hGH (1), PhoA (2), scFv Herceptin (3), scFv 3M80 (4), scFv 3211 (5), B4GalT1 (6), Herceptin Fab (7), Maa48 Fab (8), Angiopoietin-2 (9), and Avidin (10).

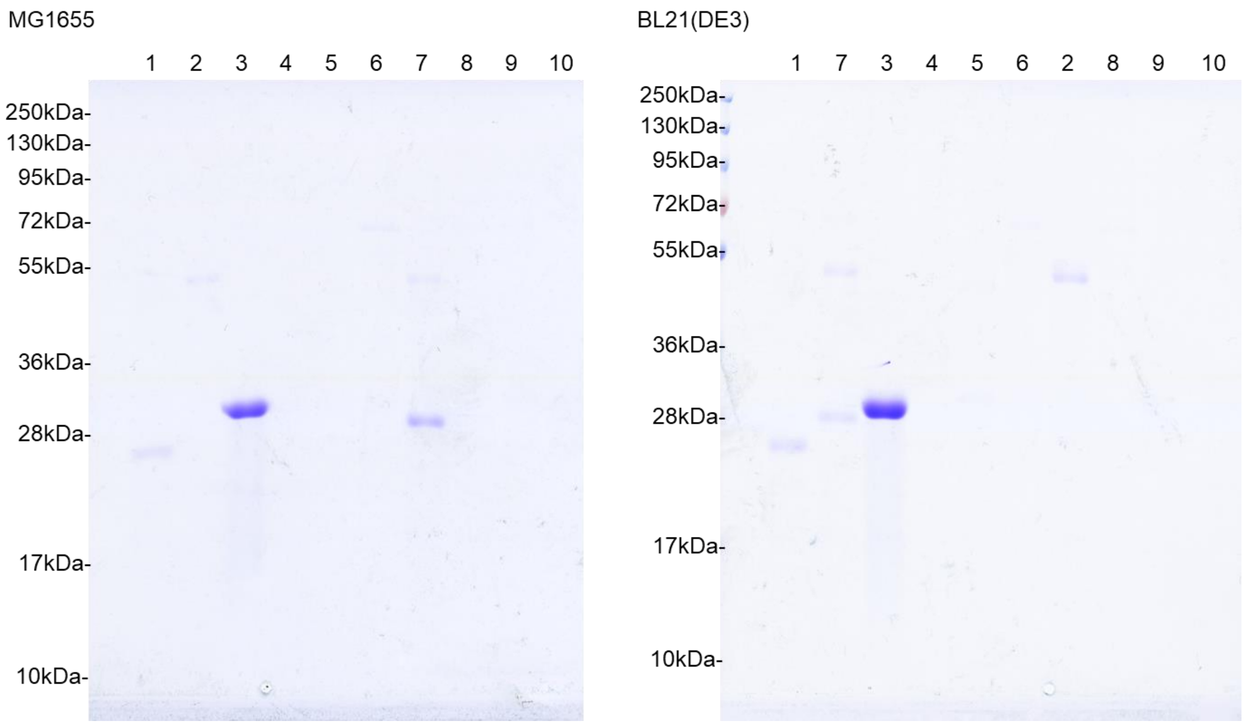

Supplementary figure S2. Coomassie stained SDS-PAGE of POI expressed in B-strains, both SHuffle and CyDisCo, using rich media. Non-reducing gel. Strains used: SHuffle T7 Express (SH) and BL21(DE3) + CyDisCo (Cy). Expressed using rich autoinduction media and Ptac promoter.

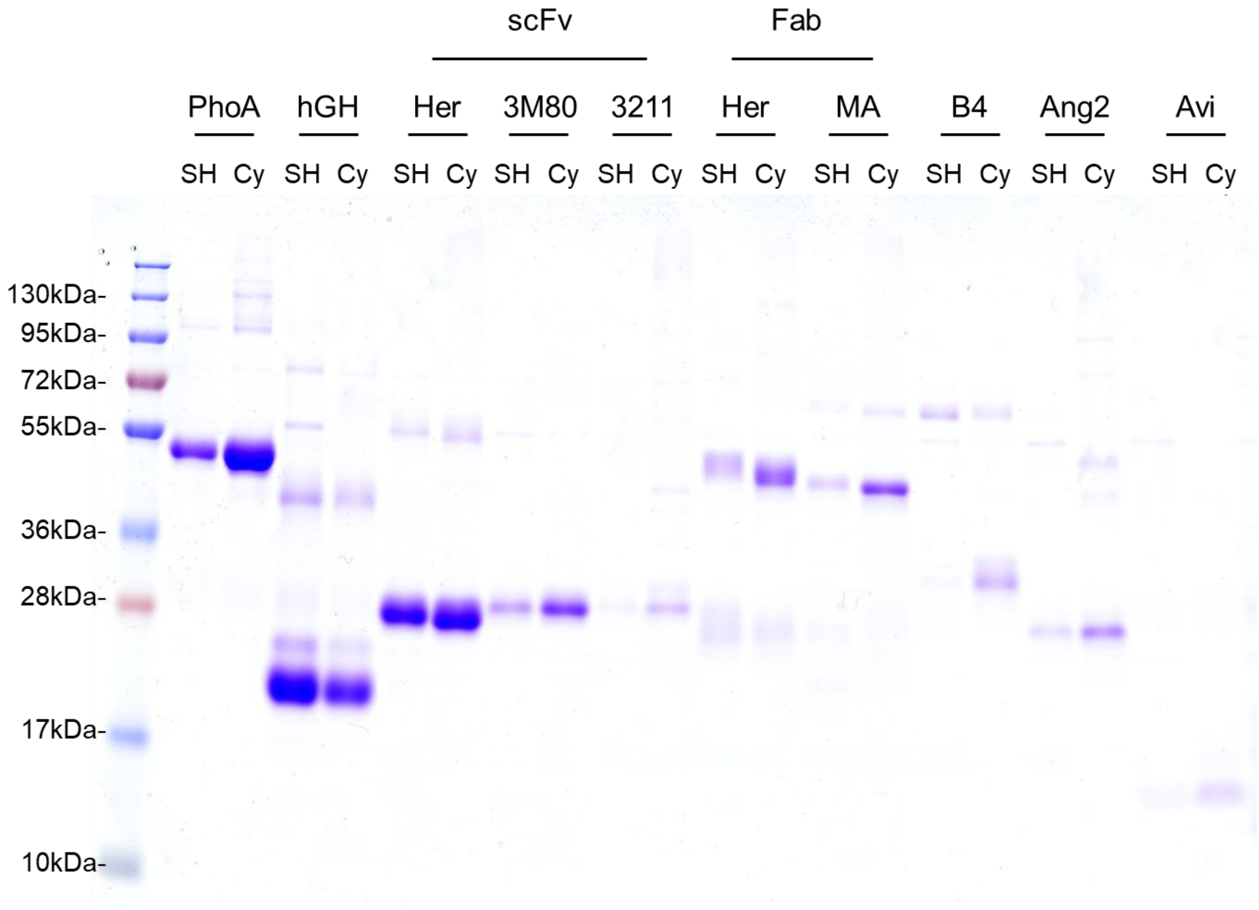

Supplementary figure S3. Coomassie stained SDS-PAGE of POI expressed in K-12 strains, both SHuffle and CyDisCo, using rich media. Non-reducing gel. The strains used were SHuffle T7 (SH) and MG1655 + CyDisCo (Cy). Expressed using rich autoinduction media and Ptac promoter.

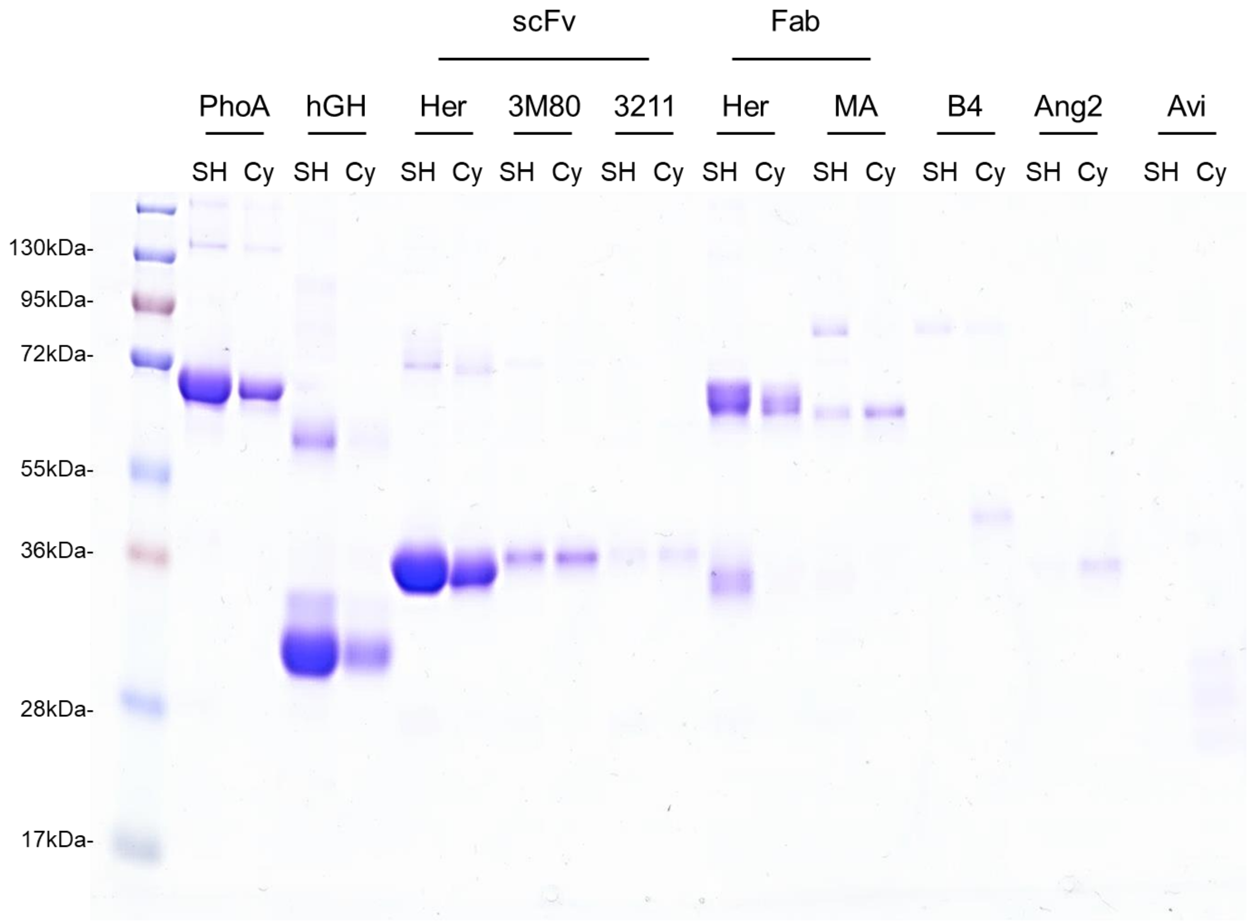

Supplementary figure S4. Coomassie stained SDS-PAGE of POI expressed in B strains, both SHuffle and CyDisCo, using defined media. Non-reducing gel. The strains used were SHuffle T7 express (SH) and BL21(DE3)+ CyDisCo (Cy) expressed using defined autoinduction media and Ptac promoter.

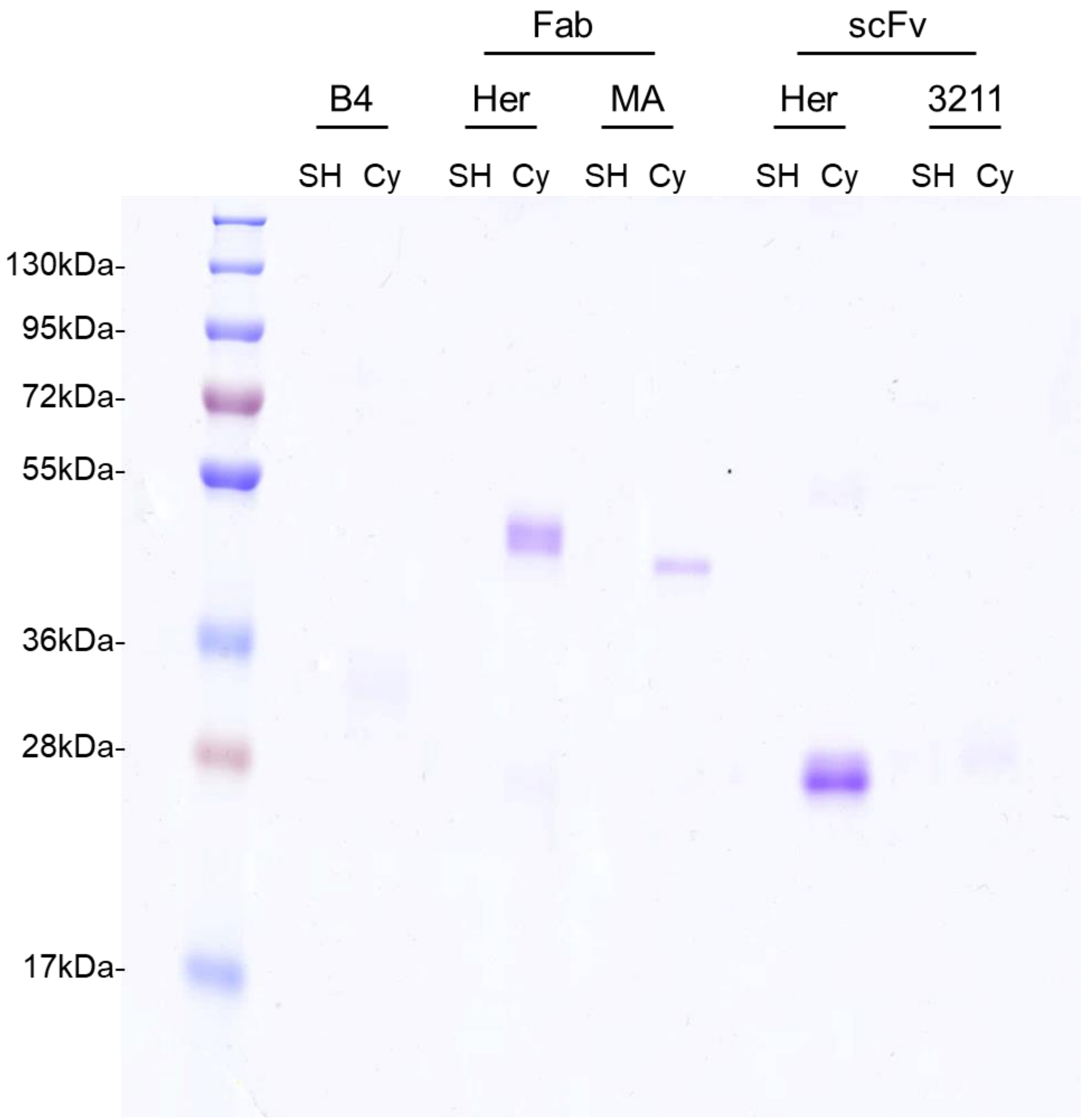

Supplementary figure S5. Coomassie stained non-reducing SDS-PAGE of POI expressed using T7 promoter in B strains, both SHuffle and CyDisCo. The strains used were SHuffle T7 express (SH) and BL21(DE3) + CyDisCo (Cy) expressed in either rich autoinduction media or defined autoinduction media.

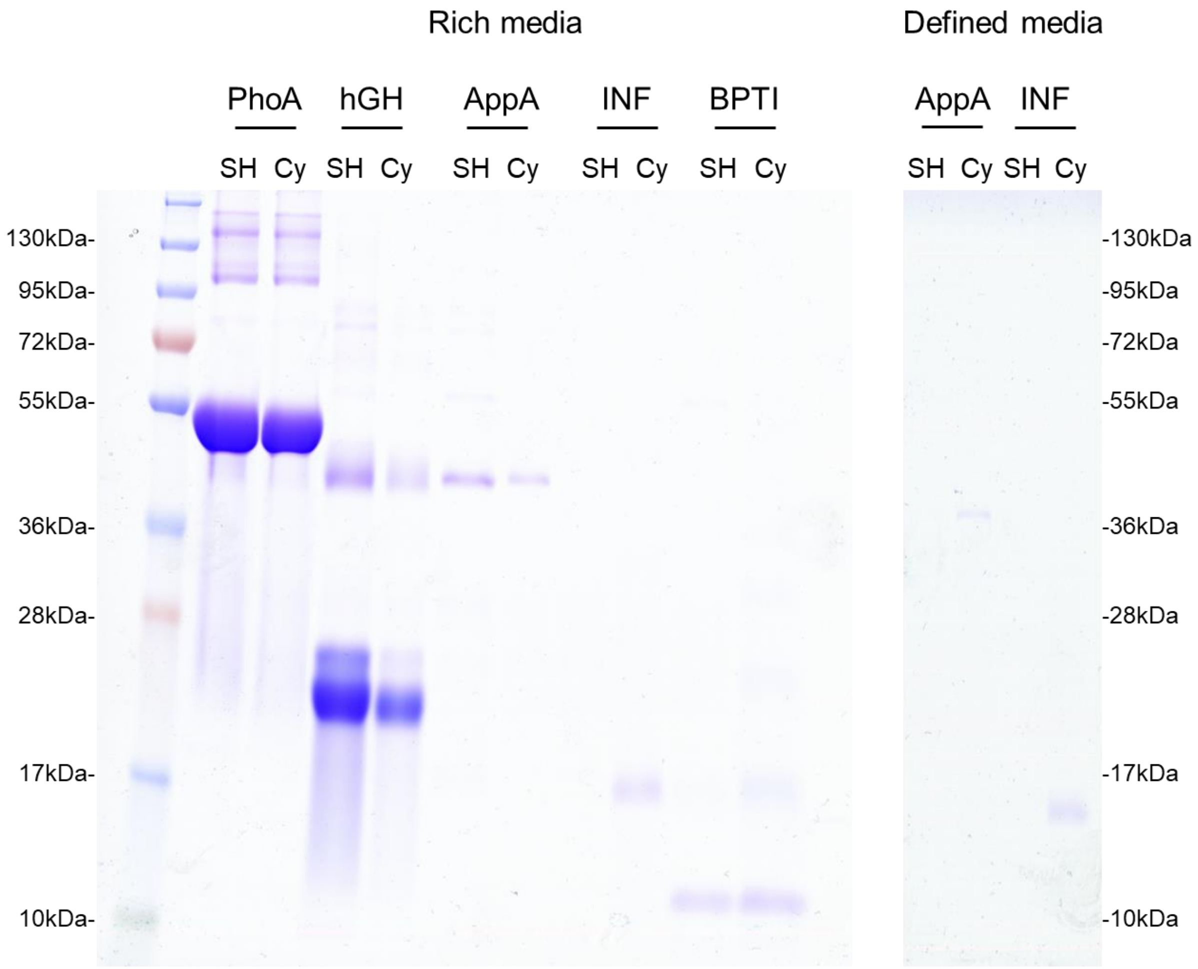

Supplementary Figure 6. Original SDS-PAGE quality of the purified scFv Herceptin and Maa48 Fab expressed under different conditions. SDS-PAGE of scFv Herceptin (A) and Maa48 Fab (B) expressed in either BL21(DE3)+CyDisCo (1), SHuffle T7 Express (2), MG1655+CyDisCo (3) or SHuffle T7 (4). Samples are analyzed as untreated under reducing conditions and as pretreated either with N-Ethylmaleimide or MalPEG5000 under non-reducing conditions (NR+NEM or NR+MalPEG, respectively).

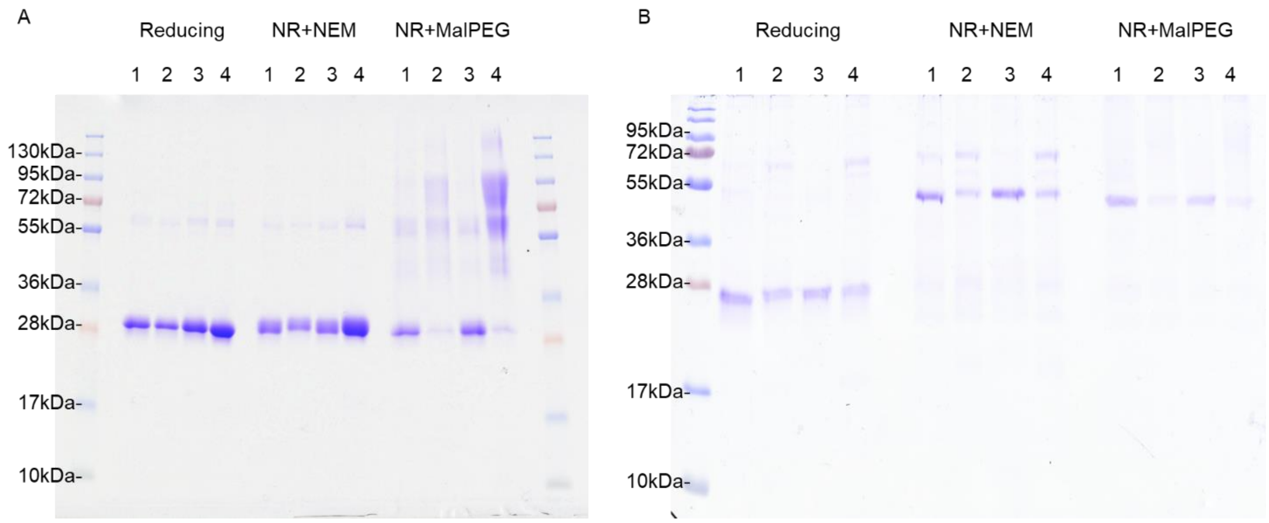

Supplementary figure S7. Coomassie stained SDS-PAGE of B4GalT1 protein expressed in rich media using either B or K-12 strains. Non reducing gel. The strains used were: SHuffle T7 express and BL21(DE3)+ CyDisCo for B strain; and SHuffle T7 and MG1655 + CyDisCo for K-12 strain. From three independent experiments. Red rectangle = the coeluted contaminant identified, blue rectangle = the POI identified.

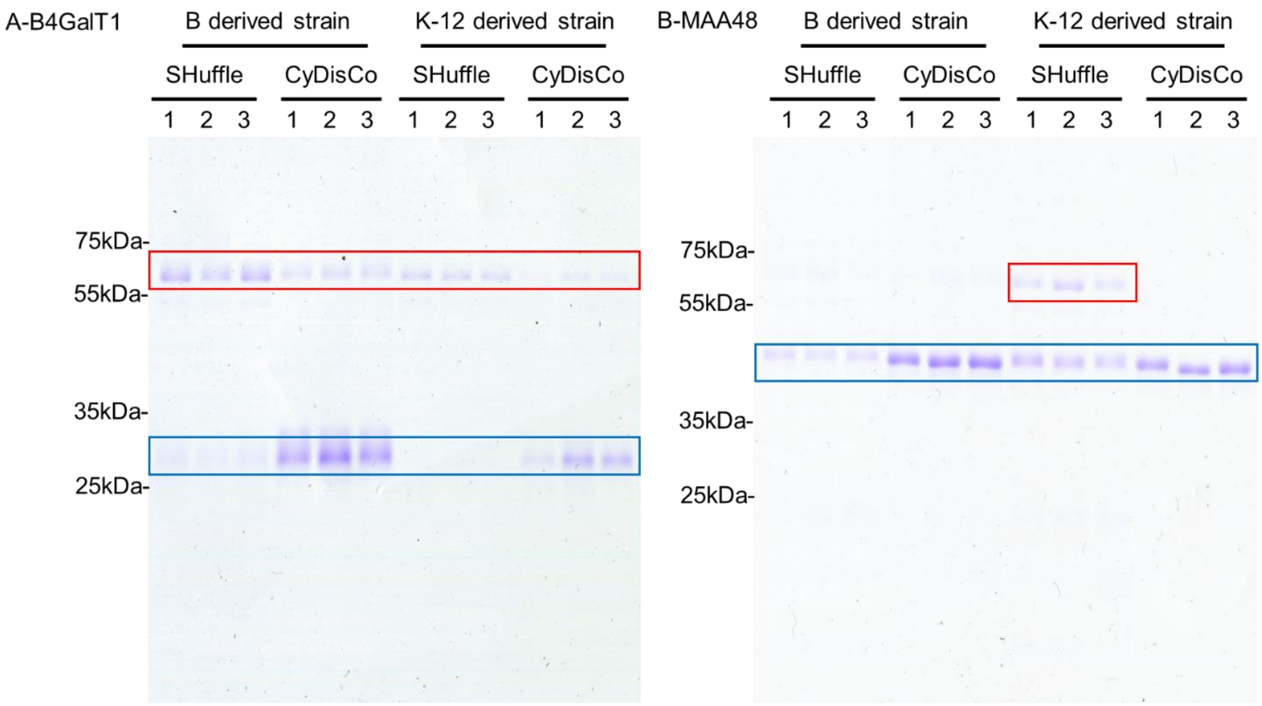

Supplementary figure S8. Coomassie stained non-reducing SDS-PAGE of Gaussia luciferase (GLuc) expressed in B strains, both Shuffle and CyDisCo. The strains used were SHuffle T7 Express and BL21(DE3) + CyDisCo in either defined autoinduction media or rich autoinduction media, under a Ptac promoter. From three independent experiments.

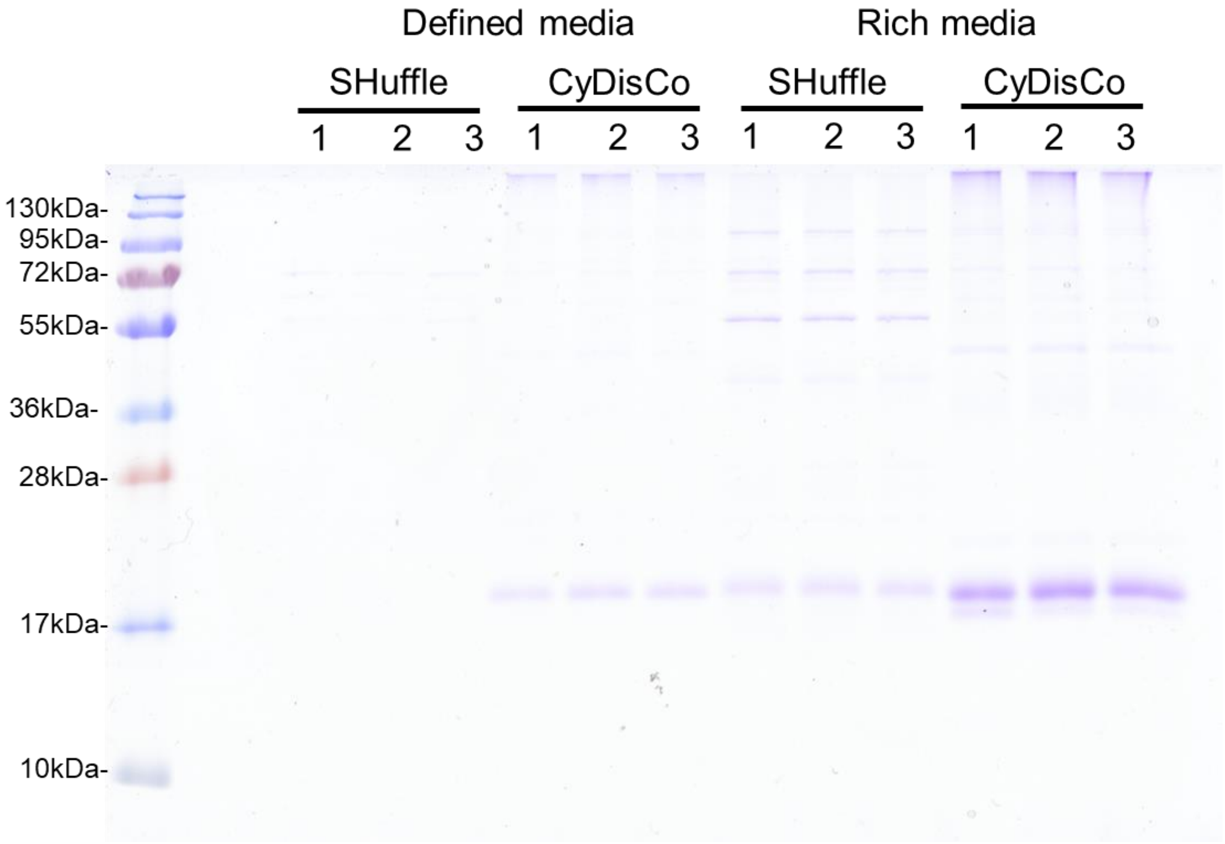

Supplementary table S1. Bacterial strains used in this study.

Name	Details	Source
BL21(DE3)	*fhuA2 [lon] ompT gal (λ DE3) [dcm]* Δ*hsdS* *λ DE3 = λ sBamHIo* Δ*EcoRI-B* *int::(lacI::PlacUV5::T7 gene1) i21* Δ*nin5*	Novagen
MG1655 (STL242)	F^{-} λ^{-} *ilvG*$^{-}$ rfb-*50 rph-1*	S. T. Lovett
SHuffle T7 Express	*fhuA2 lacZ::T7 gene1 [lon] ompT ahpC gal λatt::pNEB3-r1-cDsbC* ($Spec^{R}$, *lacI*^{q}) *ΔtrxB sulA11 R(mcr-73::miniTn10*--Tet^{S}*)2 [dcm] R(zgb-210::Tn10* --Tet^{S}*) endA1 Δgor Δ(mcrC-mrr)114::IS10*	NEB
SHuffle T7	F´ *lac, pro, lacI*^{q} */ Δ(ara-leu)7697 araD139 fhuA2 lacZ::T7 gene1 Δ(phoA)PvuII phoR ahpC* galE (or U) galK λatt::pNEB3-r1-cDsbC* ($Spec^{R}$, *lacI*^{q}) *ΔtrxB rpsL150*(Str^{R}) *Δgor Δ(malF)3*	NEB

Supplementary table S2. Details of the plasmid vectors used in this study. co = codon optimized for *E. coli*

Plasmid	Proteins expressed	Promoter	Ref.
pVD96	*E. coli* AppA H6	T7	[1]
pGZ10	co human INF-α2 H6	T7	[2]
pHEE12	co *B.taurus* BPTI	T7	[2]
pJML4	co human Growth hormone 1	T7	This study
pMJS68	*E. coli* PhoA cytoplasmic H6	T7	This study
pYU103	co *B.taurus* BPTI R36-A93 H6 tag	Ptac	This study
pMJS79	*E. coli* PhoA cytoplasmic H6	Ptac	This study
pMJS191	co Chicken Avidin H6	Ptac	This study
pHIA525	co B4GALT1cat H6	Ptac	[3]
pMJS 226	CyDisCo components with LacI	Ptac	[4]
pMJS205	CyDisCo components	Ptac	[1]
pHIA646	co Angiopoietin-2 fibrogen domain K275-F496, H6,	Ptac	This study
pJV11	co Maa48 Fab, H6,	Ptac	[5]
pJV60	co Herceptin Fab H6	Ptac	[5]
pJV78	co scFv Herceptin H6	Ptac	[5]
pJV82	co scFv 3211 H6	Ptac	[5]
pJV84	co scFv IgA1 (3M8O) H6	Ptac	[5]
pACC1	co Gluc (*Gaussia princeps* luciferase) H6	Ptac	This study
pYU225	co Human Growth hormone-1 H6	Ptac	This study

Supplementary table S3. Summary table of POI expressed in SHuffle and CyDisCo in rich media using B strains.

Protein	Strain	Mean of OD 600	Mean of yield (mg/L)	SD of yield	n	*p-value*
hGH	SHuffle	25.81	419	103	6	3.57E-02
	CyDisCo	22.98	300	40.6	6	
PhoA	SHuffle	19.78	146	37.7	6	3.53E-05
	CyDisCo	18.86	451	75.5	6	
scFv Herceptin	SHuffle	19.79	138	15.7	6	3.23E-03
	CyDisCo	16.74	378	87.8	6	
scFv 3M80	SHuffle	17.07	44.1	14.9	4	1.46E-02
	CyDisCo	19.63	100	22.8	4	
scFv -3211	SHuffle	17.67	12.7	5.35	6	8.20E-04
	CyDisCo	18.20	29.9	7.12	6	
B4GalT1	SHuffle	21.11	12.1	2.2	6	1.27E-05
	CyDisCo	18.55	42.7	5.95	6	
Herceptin Fab	SHuffle	17.30	43.3	9.82	6	4.36E-04
	CyDisCo	17.21	82.8	14.4	6	
Maa48 Fab	SHuffle	15.81	21.3	4.67	6	8.49E-07
	CyDisCo	17.58	59.0	7.27	6	
Ang-2	SHuffle	32.75	23.9	1.35	3	4.90E-03
	CyDisCo	22.81	60.0	5.34	3	
Avidin	SHuffle	15.00	4.8	1.86	6	1.76E-03
	CyDisCo	13.82	24.1	9.84	7	

Supplementary table S4. Summary table of different POI expressed in SHuffle and CyDisCo in rich autoinduction media using K-12 strains.

Protein	Strain	Mean of OD 600	Mean of yield (mg/L)	SD of yield	n	*p-value*
hGH	SHuffle	18.8	869	188	3	2.64E-02
	CyDisCo	21.3	218	11.5	3	
PhoA	SHuffle	12.7	702	65.8	3	4.44E-04
	CyDisCo	18.5	220	42.9	3	
scFv Herceptin	SHuffle	14.4	691	50.0	3	5.49E-01
	CyDisCo	10.8	737	107	3	
scFv 3M80	SHuffle	16.3	89.1	15.8	3	5.63E-01
	CyDisCo	17.5	95.7	9.16	3	
scFv 3211	SHuffle	15.4	14.3	6.02	3	1.79E-01
	CyDisCo	18.4	21.2	1.61	3	
B4GalT1	SHuffle	18.7	5.87	4.35	3	6.35E-02
	CyDisCo	18.4	11.9	1.94	3	
Herceptin Fab	SHuffle	14.4	357	133	3	7.89E-02
	CyDisCo	10.8	127	55.1	3	
Maa48 Fab	SHuffle	26.2	26.9	5.32	3	3.95E-04
	CyDisCo	18.1	78.5	6.19	3	
Ang-2	SHuffle	18.6	8.23	5.17	3	3.35E-03
	CyDisCo	18.1	34.8	5.25	3	
Avidin	SHuffle	17.5	3.24	5.60	3	5.09E-04
	CyDisCo	17.5	39.6	9.53	5	

Supplementary table S5. Summary table of different POI expressed in the strain related assays that have increased yields in K-12 strains.

Protein	Strain	Mean of OD 600	Mean of yield (mg/L)	SD of yield	n	*p-value*
hGH	SHuffle T7 Express	25,81	419	103	6	2,03E-03
	SHuffle T7 (K-12)	18,8	869	188	3	
PhoA	SHuffle T7 Express	19,78	146	37,7	6	7,16E-07
	SHuffle T7 (K-12)	12,7	702	65,8	3	
scFv Herceptin	SHuffle T7 Express	19,79	138	15,7	6	1,81E-03
	SHuffle T7 (K-12)	14,4	691	50,0	3	
scFv Herceptin	CyDisCo + BL21(DE3)	16,74	378	87,8	6	9,97E-03
	CyDisCo + MG1655	10,8	737	107	3	
Herceptin Fab	SHuffle T7 Express	17,30	43,3	9,82	6	4,38E-04
	SHuffle T7 (K-12)	14,4	357	133	3	
Maa48 Fab	CyDisCo + BL21(DE3)	17,58	59,0	7,27	6	5,53E-03
	CyDisCo + MG1655	18,1	78,5	6,19	3	
Avidin	CyDisCo + BL21(DE3)	13,82	24,1	9,84	7	2,18E-02
	CyDisCo + MG1655	17,5	39,6	9,53	5	

Supplementary table S6. Summary table of different POI expressed in SHuffle and CyDisCo in chemically defined autoinduction media using B strains.

Protein	Strain	Mean of OD 600	Mean of yield (mg/L)	SD of yield	n	*p-value*
scFv Herceptin	SHuffle	13.9	4.92	3.3	3	1.64E-03
	CyDisCo	11.4	97.3	21	3	
scFv 3211	SHuffle	13.0	0.00	0.0	3	3.00E-05
	CyDisCo	11.8	7.03	0.6	3	
Herceptin Fab	SHuffle	14.4	0.00	0.0	3	2.20E-03
	CyDisCo	18.7	61.5	5.0	3	
Maa48 Fab	SHuffle	12.5	0.00	0.0	3	2.90E-05
	CyDisCo	11.9	13.7	1.4	3	
B4GalT1	SHuffle	13.4	0.00	0.0	3	4.49E-04
	CyDisCo	13.0	11.9	1.9	3	

Supplementary table S7. Summary table of different POI expressed in B strains, both SHuffle and CyDisCo in either rich or chemically defined autoinduction media, under a T7 promoter.

Protein	Strain	Mean of OD 600	Mean of yield (mg/L)	SD of yield	n	*p-value*
hGH	SHuffle	24.1	790	74.5	3	1.98E-03
	CyDisCo	23.1	433	43.0	3	
PhoA	SHuffle	23.3	1766	582	3	9.62E-02
	CyDisCo	29.1	904	368	3	
AppA	SHuffle	17.5	28.6	3.00	3	5.31E-03
	CyDisCo	18.1	16.3	2.44	3	
BPTI	SHuffle	15.9	67.4	36.1	6	5.31E-03
	CyDisCo	14.4	154	47.6	6	
INFα-2b	SHuffle	15.8	2.72	2.44	3	1.70E-05
	CyDisCo	15.8	33.3	6.27	6	
AppA (Defined media)	SHuffle	14.8	0.00	0.00	3	1.21E-04
	CyDisCo	12.6	1.89	0.61	5	
INFα-2b (Defined media)	SHuffle	14.4	0.00	0.00	3	4.85E-04
	CyDisCo	12.2	15.2	2.54	3	

Supplementary table S8. Summary table of different GLuc expressed in SHuffle and CyDisCo in either rich or chemically defined autoinduction media using B strains.

Media	Strain	Mean of OD 600	Mean of yield (mg/L)	SD of yield	n	*p-value*
Defined	SHuffle	13,7	0,00	0,00	3	9,69E-03
	CyDisCo	11,2	24,4	4,19	3	
Rich	SHuffle	20,7	23,4	4,37	3	3,85E-04
	CyDisCo	15,6	75,8	6,98	3	

List of abbreviations

co: codon optimized

scFv: single-chain variable fragment.

Fab: Fragment of antibody.

PhoA: *E. coli* alkaline phosphatase (PhoA).

hGH: human growth hormone (hGH).

Her: Herceptin.

MA: Maa48.

B4: human Beta-1,4-galactosyltransferase 1 (B4Galt1).

Ang2: human Angiopoietin-2 (Ang-2).

Avi: Chicken Avidin.

AppA: *E. coli* phytase (AppA).

INF: human interferon alpha 2b (IFNα-2b).

BPTI: bovine pancreatic trypsin inhibitor (BPTI).

References

1. Hatahet F, Nguyen VD, Salo KEH, Ruddock LW. 2010. Disruption of reducing pathways is not essential for efficient disulfide bond formation in the cytoplasm of E. coli. *Microb. Cell Fact.* **9**: 67–75.

2. Nguyen VD, Hatahet F, Salo KEH, Enlund E, Zhang C, Ruddock LW. 2011. Pre-expression of a sulfhydryl oxidase significantly increases the yields of eukaryotic disulfide bond containing proteins expressed in the cytoplasm of E.coli. *Microb. Cell Fact.* **10**: 1–13.

3. Harrus D, Khoder-Agha F, Peltoniemi M, Hassinen A, Ruddock L, Kellokumpu S, *et al.* 2018. The dimeric structure of wild-type human glycosyltransferase B4GalT1. *PLoS One* **13**: e0205571.

4. Coker JA, Marzeda A, Schwenzer A, Marsden BD, Midwood KS, Yue WW. 2021. *Fibrinogen-like globe domain of human Tenascin-C (hFBG-C); A Target Enabling Package*. Zenodo.

5. Gaciarz A, Veijola J, Uchida Y, Saaranen MJ, Wang C, Hörkkö S, *et al.* 2016. Systematic screening of soluble expression of antibody fragments in the cytoplasm of E. coli. *Microb. Cell Fact.* **15**: 1–10.
